# Homeopathic Treatment of *Arabidopsis thaliana* Plants Infected with *Pseudomonas syringae*

**DOI:** 10.1100/tsw.2009.38

**Published:** 2009-05-20

**Authors:** Devika Shah-Rossi, Peter Heusser, Stephan Baumgartner

**Affiliations:** ^1^ Society for Cancer Research, Hiscia Institute, Kirschweg 9, 4144 Arlesheim, Switzerland; ^2^ Institute of Complementary Medicine KIKOM, University of Bern, Insel-Spital, Imhoof-Pavillon, 3010 Bern, Switzerland

**Keywords:** Pseudomonas syringae, plant disease, systemic acquired resistance (SAR), homeopathy

## Abstract

Homeopathic basic research is still in the screening phase to identify promising model systems that are adapted to the needs and peculiarities of homeopathic medicine and pharmacy. We investigated the potential of a common plant-pathogen system, Arabidopsis thaliana infected with the virulent bacteria Pseudomonas syringae, regarding its response towards a homeopathic treatment. A. thaliana plants were treated with homeopathic preparations before and after infection. Outcome measure was the number of P. syringae bacteria in the leaves of A. thaliana, assessed in randomized and blinded experiments. After a screening of 30 homeopathic preparations, we investigated the effect of Carbo vegetabilis 30x, Magnesium phosphoricum 30x, Nosode 30x, Biplantol (a homeopathic complex remedy), and Biplantol 30x on the infection rate in five or six independent experiments in total. The screening yielded significant effects for four out of 30 tested preparations. In the repeated experimental series, only the homeopathic complex remedy Biplantol induced a significant reduction of the infection rate (p = 0.01; effect size, d = 0.38). None of the other four repeatedly tested preparations (Carbo vegetabilis 30x, Magnesium phosphoricum 30x, Nosode 30x, Biplantol 30x) yielded significant effects in the overall evaluation. This phytopathological model yielded a small to medium effect size and thus might be of interest for homeopathic basic research after further improvement. Compared to Bion (a common SAR inducer used as positive control), the magnitude of the treatment effect of Biplantol was about 50%. Thus, homeopathic formulations might have a potential for the treatment of plant diseases after further optimization. However, the ecological impact should be investigated more closely before widespread application.

## INTRODUCTION

Homeopathy is still a controversial field, although many clinical and experimental research projects have been carried out to date. Astonishingly, to our knowledge, nearly all quantitative meta-analyses of randomized clinical trials for specific medical conditions yielded positive evidence for homeopathic treatments compared to placebo[1,2,3,4,5,6]. These results imply a specific drug action for homeopathic preparations and, correspondingly, some sort of "memory" of the homeopathic carrier material (water, ethanol, lactose) for the substance potentized.

Also, in preclinical research, several meta-analyses concluded that there is some evidence for specific homeopathic remedy effects[7,8,9]. The main problem in this research field, however, seems to be the reproducibility of the results obtained[10]. It is under current investigation whether this phenomenon is of intrinsic nature (as in, for example, chaos theory) or whether it is due to a lack of knowledge of relevant parameters that influence the experimental system.

One hypothesis in this context is that the state of the experimental system is crucial for a reproducible reaction to homeopathic preparations. The information furnished by the homeopathic preparation has to be meaningful for the experimental system and this depends on the state of the system[11]. Thus, it may seem plausible that diseased organisms may be more adequate experimental systems than healthy organisms in order to identify specific effects of homeopathic preparations. Diseased organisms can be seen to be in a state out of equilibrium, with the innate tendency to return to the equilibrium state called health.

As reviewed elsewhere[8,12], there were many attempts to disturb biological model systems by the introduction of toxic compounds (e.g., arsenic, copper sulfate) and there is indeed some evidence that poisoned organisms react more strongly to homeopathic preparations than healthy ones[13]. However, we know of only a few attempts to investigate the potential of homeopathic preparations in diseased plants, i.e., in phytopathological models[8]. We thus decided to investigate a common and comparably simple phytopathological system regarding its potential for homeopathic basic research: *Arabidopsis thaliana* infected with virulent bacteria from the species *Pseudomonas syringae (*pv *tomato* strain DC3000). This system was chosen for several reasons. It is well known regarding plant-bacteria interactions and is an ideal model system for studies on host defense responses to pathogen attack[14], thus providing the option to investigate the mode of action of homeopathic preparations on plants in case that positive effects of homeopathic dilutions can be identified. Even though the small cruciferous plant *A. thaliana* has no agroeconomic relevance, it often serves as a study model because of its experimental advantages (small size, short life cycle, self-fertilizing property, etc.).

Besides being of interest for homeopathic basic research, positive results might motivate further investigations in order to reveal the potential of homeopathic approaches for plant protection in agriculture since a homeopathic treatment can be hypothesized to have fewer ecological side effects on nontarget organisms than some standard treatments because of the absence of harmful substantial doses of various chemicals.

In principle, one could imagine that homeopathic preparations are able to exert either a prophylactic activity (and, for example, induce resistance to bacterial disease in plants, the so-called systemic acquired resistance[15] or SAR) and/or a therapeutic activity by curing a bacterial infection within a plant. Even though a prophylactic application of a homeopathic remedy does not necessarily reveal a therapeutic activity, we decided to treat the plants always both before and after the infection in order to maximize any effect of the homeopathic preparations. Outcome measure was the number of bacteria in the leaves. We first performed a screening to identify potentially effective homeopathic preparations. The screening comprised classical homeopathic remedies, potentized metals, a nosode, potentized salicylic acid, and a commercially available homeopathic complex remedy. The most promising candidates from the screening were then tested in four or five further independent experiments to assess treatment effects reliably.

## MATERIALS AND METHODS

The methodology was adapted from Dong et al.[16], Whalen et al.[17], and personal communication from Dr. B. Mauch-Mani, affiliated at Fribourg University, Switzerland, during the experimental phase of the present investigation.

## Cultivation of Plants

*Arabidopsis thaliana* seeds (Col-0, wild-type, from Lehle Seeds, Round Rock, Texas) were sown on a mixture of sterile potting soil (TKS1 ready-to-use growing medium based on slightly decomposed peat, from Floragard GmbH, Oldenburg, Germany) and sand. After 1 month, they were transplanted singly into small pots (20 ml) containing an analogous mixture of TKS1 and sand. Each pot was covered with nylon net with a small opening through which the seedling developed. This procedure enabled the plant to be dipped upside down into the bacterial suspension for infection and avoided any contamination of the suspension with soil. The plants were grown under artificial light (with supplementary fluorescent lighting, 90 ± 10 µmol photons $m^{-2}sec^{-1}$ PAR [photosynthetically active radiation]) with an 8-h photoperiod at 25 ± 2°C. No additional fertilizer was used. Plants were watered using tap water twice a week. The geographic location of the experiments performed was Arlesheim, Switzerland.

## Selection of Homeopathic Substances

The selection of homeopathic substances for the initial screening was based on different approaches. In classical homeopathy, remedies are chosen according to the symptoms and their modalities experienced by the patient. We tried to adapt this approach to our plant model and selected remedies as listed in the human Materia Medica, with the assistance of a homeopath (M. Frei, MD, KIKOM, University of Bern, Switzerland), by extrapolating from human symptoms and organs to plant symptoms and organs. The diseased leaves of the plants were considered as skin (with inflamed, dry, burned, necrotic, or gangrenous skin; with chicken pox or measles) or as lungs (pneumonia, breathing problems). The choice was then refined according to environmental factors worsening or improving the infection (modalities). It appeared that the symptoms of *A. thaliana* infected with *P. syringae* improved in well-lit, dry, cool places, but worsened in dark and humid places. We therefore selected the preparations Antimonium metallicum, Argentum nitricum, Arsenicum album, Carbo animalis, Carbo vegetabilis, Carbolicum acidum, Crotalus horridus, Equisetum, Fluoricum acidum, Kreosotum, Lachesis, Lycopodium, Phosphorus, Rhus toxicodendron, Secale cornutum, Silicicum acidum, Sulfur, and Tabacum.

A second approach involved systemic acquired resistance (SAR). SAR is a kind of “induced plant immunity” that acts nonspecifically throughout the plant and reduces the severity of disease caused by a broad range of pathogens[18]. SAR can be triggered in response to infection of the plant with a pathogen, or through certain natural or synthetic chemical compounds, such as salicylic acid, arachidonic acid, or 2,6-dichloro-isonicotinic acid (INA), applied through the roots, as a foliar spray or by stem injection[19]. We therefore tested potentized salicylic acid.

A third approach was the use of a nosode: a potentized extract of infected plant tissue.

The fourth approach was to test different metals such as Aurum colloidale, Calcium carbonicum, Cuprum sulfuricum, Ferrum metallicum, Kalium iodatum, Magnesium phosphoricum, Plumbum metallicum, and Zincum sulfuricum, as some metals play an important role in plant nutrition and their resistance to disease[20].

Additionally, a commercially available complex homeopathic preparation specially made for plants (Biplantol SOS®, Bioplant Naturverfahren GmbH, Konstanz, Germany) was tested. This product is composed of a mixture of different nutrients in potentized form (N, P, K, Mg, Ca, S, Fe, Cu, Co, Mn, B, Zn, V, Mo, Si, Ge, and uronic acid, all in potency levels between 6x and 200x).

## Preparation of Homeopathic Potencies and Controls

The homeopathic preparations used were applied at the potency level 30x (D30). Biplantol only was also tested in its original formulation (not additionally potentized). For the control group, sterile purified succussed water (1x) was used in order to exclude effects from unspecific physicochemical alterations that may occur due to the succussion procedure (for a detailed discussion of useful controls in homeopathic basic research see Baumgartner et al.[21]).

Homeopathic preparations (except for the nosode and Biplantol) were provided by Phytomed AG (Hasle/Burgdorf, Switzerland) and consisted of potencies between 1x and 9x potentized in 43% (w/w) ethanol. All potencies were potentized up to potency level 9x in ethanol 43% (1 ml of the previous homeopathic preparation with 9 ml of ethanol 43% succussed for 1 min) to achieve comparable conditions regarding ethanol content. Potencies 10x up to 29x were made in sterile purified water (1 ml of the previous homeopathic preparation in 9 ml sterile purified water, succussed analogously) in 20-ml disposable PP-tubes with PE-caps (Semadeni: no. 3188 and 1467, Ostermundigen, Switzerland). Potency level 30x (4-ml preparation in 36-ml sterile purified water) was made in 50-ml disposable PP-tubes (Polylabo: no. 430829, Genève, Switzerland). The water used for potentization was tap water purified through the Millipore Elix system, consisting of purification by reverse osmosis and then electrodeionization, resulting in 15 MΩcm water. All potencies were potentized for 1 min by hand, with a moderate, regular, rhythmic, up-down agitation, without using a hard surface as support in the process.

As the composition of Biplantol is only partially disclosed, it was not only applied in its commercially available form, but also potentized up to 30x. The potentization from 1x up to 29x and 30x were made in sterile purified water as described above.

Salicylic acid was obtained from Pharmacie du Lion d'Or (Lausanne, Switzerland).

The nosode preparation was freshly made for each experiment as follows: 40 leaves of untreated infected *A. thaliana* plants (see below) were mixed with 40 leaves of healthy *A. thaliana* plants (according to a suggestion of M. Bastide, personal communication), and ground for 5 min in a mortar with 1 g of silica sand and 3 ml of sterile purified water. The extract was filtered through a piece of cotton wool. This extract was defined as 0x preparation, which was then potentized with sterile purified water up to 29x and 30x as described above.

All aqueous homeopathic preparations and controls were freshly prepared on the day of pretreatment of the plants (see below).

## Pretreatment of the Plants with Homeopathic Potencies

For the experiments, *A. thaliana* plants were used at an age of 2–3 months, i.e., when exhibiting large leaves (1–2 cm long), but before the appearance of flowering shoots. Plants were fully plunged upside down for 30 sec into 20 ml of the homeopathic preparation or the control solution, to which a surfactant (Silwet L-77, from Lehle Seeds) was added at a concentration of 0.01% (v/v). This method is a standard method in phytopathology for artificial infection of plants by means of the stomata (see below). We therefore assumed that the homeopathic remedies applied in this way would also penetrate the plants.

In order to optimize the uptake of homeopathic preparations, 1.5 ml of the dipping solution was dropped into the center of the rosette of each plant, from where it leaked into the soil, and the remaining solution was added at the same time to the irrigation water. The plants were covered with a plastic foil to keep them at a high degree of humidity until the moment of the infection, occurring 2 days later (see below).

The plants in this project were treated with the homeopathic preparations or controls during the hours of darkness, i.e., between 9 and 11 p.m. This was based on the chronobiological findings of Rikin and Anderson, who observed that cotton plants (*Gossypium hirsutum)* present daily changes in their sensitivity to herbicides; maximal sensitivity to the herbicides was 4 to 8 h after the onset of darkness[22]. In a review on chronophytopathology, Kennedy and Koukkari cite results of two other research projects in which the application of pesticides (streptomycin for fire blight disease in orchards, and a herbicide) was greatly enhanced when applied at night instead of in daylight[23].

## Infection of the Plants

The bacteria *P. syringae (*pv *tomato* strain DC3000, obtained from the Department of Plant Biology at Fribourg University) was maintained at –80°C in a sterile mixture of 0.85-ml King's Medium B and 0.15-ml glycerol. One day after pretreatment of the plants (i.e., 1 day before infection), a culture of *P. syringae* in King's Medium B + Rifampicine (50 µg/ml) at 28°C and 100 rpm was started. After about 18 h of growth, the cells were centrifuged (at 4000 rpm), washed in sterile $MgSO_4$ 0.01 *M*, and diluted in sterile $MgSO_4$ 0.01 *M* in order to obtain a concentration of the suspension of $OD_{600}$ = 0.1 (measured spectrophotometrically). This corresponded approximately to a concentration of $10^8$ cfu (colony forming units)/ml. The bacterial suspension was finally diluted to $10^5$ cfu/ml in $MgS0_4$ 0.01 *M*. A surfactant (Silwet L-77) was added to the bacterial suspension at a concentration of 0.01% to enable the bacterial suspension to penetrate the leaves via the stomata. The *A. thaliana* plants were fully plunged upside down for 30 sec into 20 ml of this bacterial suspension. Plants with different homeopathic pretreatments or control treatment were dipped into different bacterial containers to avoid possible cross-contamination. Subsequently, the plants were covered with plastic foil and again kept at a high humidity for 4 days in order to promote a thorough infection.

## Post-Treatment of the Plants with Homeopathic Potencies

One day after infection, the remainder of the homeopathic potencies used for pretreatment (20 ml, conserved at 4°C) was administered to the plants. Because the leaves were full of bacteria, the dipping method was no longer possible. Therefore, 1 ml of the homeopathic solution was dropped into the center of the rosette of each plant, from where it leaked into the soil, and the remaining solution (7–12 ml, depending on the number of plants in the experiment) was added at the same time to the irrigation water. No post-treatment was done in experiment no. 21 (nosode 30x) and in experiment no. 40 (Bion®, 0.2 mg/ml, see below).

## Degree of Bacterial Infection in Leaves

The rate of infection in the leaves was determined through bacterial growth quantification after 4 days of infection. For each plant, the six biggest leaves were selected, and from each leaf a disc was sectioned with a cork borer (0.5 $cm^2$) on a piece of sterile silicon. The six discs of each plant were gathered into a 1.5-ml Eppendorf tube containing 150-µl $MgSO_4$ 0.01 *M*. The bacteria were extracted out of the leaf tissue by macerating and homogenizing the leaf discs with a glass pestle fitting the Eppendorf tube. $MgSO_4$ 0.01 *M* (1050 µl) was added to the homogenate, and serial dilutions (in $MgS0_4$ 0.01 *M*) of this extract were plated onto fresh King's Medium B plates containing rifampicine (50 µg/ml). Since *P. syringae* is resistant to rifampicine, other bacteria could be impeded from growing on the plates, thus allowing exact determination of the number of *P. syringae* colonies. The number of colonies was counted after 7 days of incubation at room temperature, and the number of colony forming units per leaf disc was calculated.

## General Experimental Design, Statistics, and Treatment of Data

Each experiment consisted of one set of eight to 13 plants pre- and post-treated with water 1x (= control group), and one, two, or three sets of eight to 13 plants treated with homeopathic preparations. The screening encompassed 30 homeopathic preparations. From the screening, five preparations were selected for the main experiments: Carbo vegetabilis 30x, Magnesium phosphoricum 30x, Nosode 30x, Biplantol

30x, and Biplantol in original formulation. Each of these five preparations was tested in four or five additional independent experiments.

Besides the experiments with the homeopathic preparations, three control experiments were carried out: two negative control experiments (water and ethanol 43% 21x) as well as a positive control experiment with Bion (0.2 mg/ml), a commercially available chemical able to induce SAR in plants (Novartis, Switzerland). The latter approach allows testing of the experimental system's sensitivity to established SAR inducers.

Except for experiment no. 21, all treatments were coded (blind experiments).

All data were analyzed with the statistics software "Statistica 4.1" (Statsoft, Inc., Tulsa, Oklahoma). In order to achieve a normal data distribution, the number of colony forming units of *P. syringae* per leaf disc was logarithmized for further statistical analysis. Thus, the outcome measure (dependent variable) was the number of grown *P. syringae* bacteria (log (cfu/0.5 $cm^2$ leaf area)) in the leaves of *A. thaliana* plants.

Data of the control experiments were analyzed with the t-test for independent samples. The data of the screening experiments were normalized to the water control of each experiment and then analyzed jointly in a one-way analysis of variance (ANOVA). Effects of the treatments in the main experiments were statistically analyzed by a two-way ANOVA, comprising treatment and experiment number (date) as the two independent variables (factors) and the number of bacteria (log (cfu/0.5 $cm^2$)) as dependent variables. Planned comparisons were evaluated with the LSD test only if the preceding F test was significant ($p < 0.05$). This procedure (protected Fisher's LSD) gives a good safeguard against type I error without being too conservative, i.e., it also gives good security against type II error[24].

## RESULTS AND DISCUSSION

### Control Experiments

The positive control experiment with Bion yielded a clear difference between treatment and control group (Table 1). Thus, the chosen experimental system is suited to detect effects of substances that are known to induce SAR against bacterial infections.

The two negative control experiments yielded no significant differences between the treatment groups (Table 1). First we tested whether the change in potentization medium at the potency level 9x, where ethanol 43% was replaced by water, had any effect on the infection rate. However, application of ethanol 21x (potentized in water) did not differ from water 1x. Second, a single systematic negative control experiment comparing two identically treated water control groups with n = 12 plants each did not yield evidence for systematic errors within the experimental setup chosen.

### Screening

Table 2 lists all 30 tested homeopathic preparations as well as their effect on the infection rate of *A. thaliana* (each value was normalized to the respective control group set to 1) and the corresponding *p* value (LSD test, comparison with the plants of the control groups treated with water 1x, pooled from all experiments). Some homeopathic preparations yielded statistically significant results: Biplantol (in original formulation), Carbo vegetabilis 30x, Sulfur 30x, and Magnesium phosphoricum 30x (Table 2). The first three remedies seemed to reduce the infection in *A. thaliana*, whereas Magnesium phosphoricum 30x seemed to increase the number of the bacterium in the plant. Since the LSD test does not correct for multiple testing, some of these significant results could be due to chance only.

**TABLE 1**
**Results of the Positive and Negative Control Experiments**

| | Tested Preparation (Mean ± SD) | Control Sample (Mean ± SD) | t-Test (df) | t-Test (*p*) |
|---|---|---|---|---|
| Positive control | Bion (0.2 mg/ml) | Water 1x | | |
| Log (cfu/0.5 cm$^2$) | 3.57 ± 0.45 | 4.88 ± 0.45 | 10 | <0.001 |
| Negative control | Ethanol 21x | Water 1x | | |
| Log (cfu/0.5 cm$^2$) | 4.77 ± 0.90 | 5.19 ± 0.50 | 16 | 0.235 |
| Negative control | Water 1x | Water 1x | | |
| Log (cfu/0.5 cm$^2$) | 4.33 ± 1.05 | 4.36 ± 0.85 | 22 | 0.940 |

Logarithm of the number of colony forming units (cfu) of *P. syringae* bacteria per 0.5 cm$^2$ leaf area of *A. thaliana* (mean ± standard deviation [SD]). Statistical evaluation by t-test (df = degrees of freedom).

## Main Experiments

In order to gain information about consistency and reproducibility for five preparations, we performed four or five additional independent experiments each: Carbo vegetabilis 30x, Magnesium phosphoricum 30x, Nosode 30x, Biplantol 30x, and Biplantol (in original formulation). For the final statistical evaluation, the data from the screening experiments were added to these further experiments.

ANOVA yielded significant effects for a treatment with Biplantol (original formulation) only (Table 3, Fig. 1E). All other tested preparations had no significant treatment effect. Since the interactions were not significant ($p > 0.15$, Table 3), there is no evidence for a strong dependency of the treatment effects on any factors associated with the single experiments. On average, a treatment with Biplantol reduced the bacterial infection of *A. thaliana* from 4.32 to 3.78 log (cfu/0.5 cm$^2$ leaf area), i.e., by 12.5% (effect size, d = 0.38). This is about half the effect that can be achieved with Bion (0.2 mg/ml, Table 1).

Application of Biplantol further potentized up to 30x did not yield significant effects (Table 3, Fig. 1D). On average, the treatment with Biplantol 30x reduced the bacterial infection from 4.75 to 4.48 log (cfu/0.5 cm$^2$ leaf area), i.e., by 5.7%. It thus does not seem to be promising to apply Biplantol at a potency level of 30x.

Comparably, Carbo vegetabilis 30x, Magnesium phosphoricum 30x, and Nosode 30x do not seem to be promising candidates for further evaluation since the initially interesting results of the screening could not be confirmed in the main experimental series (Figs. 1A–C).

Thus, in our study, a complex remedy was more successful than all tested single remedies. This result could be due to the fact that we were not able to identify the appropriate simile correctly. Furthermore, potency levels other than 30x might be more effective for plant treatment[25,26,27,28,29,30].

To our knowledge, this investigation is the first to use bacteria as pathogen in a phytopathological model system; most studies used fungi, viruses, or nematodes as pathogens[8]. Furthermore, we do not know any peer-reviewed study that investigated Biplantol. Thus, direct comparison of our results to other studies is not feasible. There are some investigations, however, which provide some evidence for beneficial effects of homeopathic preparations in other phytopathological models[8].

The exact composition of Biplantol SOS is not known; according to our information, the product is composed of a mixture of different nutrients in potentized form (N, P, K, Mg, Ca, S, Fe, Cu, Co, Mn, B, Zn, V, Mo, Si, Ge, and uronic acid, all in potency levels between 6x and 200x). Scientific progress would be expedited if the exact composition would be disclosed to the public.

**TABLE 2**
**Results of the Screening**

| Preparation | Exp. No. | N | Log (cfu/0.5 cm$^2$) | SD | LSD Test (*p*) |
|---|---|---|---|---|---|
| Antimonium metallicum 30x | 61 | 10 | 0.96 | 0.14 | 0.606 |
| Argentum nitricum 30x | 38 | 9 | 1.01 | 0.17 | 0.893 |
| Arsenicum album 30x | 52 | 10 | 0.93 | 0.29 | 0.392 |
| Aurum colloidale 30x | 60 | 10 | 0.85 | 0.36 | 0.065 |
| Biplantol SOS 30x | 44 | 11 | 0.88 | 0.13 | 0.125 |
| Biplantol SOS (original formulation) | 44 | 11 | **0.83** | 0.19 | **0.026** |
| Calcium carbonicum 30x | 58 | 10 | 1.02 | 0.20 | 0.770 |
| Carbolicum acidum 30x | 54 | 10 | 1.11 | 0.36 | 0.153 |
| Carbo animalis 30x | 54 | 10 | 1.06 | 0.40 | 0.491 |
| Carbo vegetabilis 30x | 52 | 10 | **0.79** | 0.23 | **0.010** |
| Crotalus horridus 30x | 61 | 10 | 1.02 | 0.06 | 0.839 |
| Cuprum sulfuricum 30x | 58 | 10 | 1.00 | 0.23 | 0.985 |
| Equisetum 30x | 39 | 9 | 0.99 | 0.17 | 0.933 |
| Ferrum metallicum 30x | 60 | 10 | 0.89 | 0.32 | 0.167 |
| Fluoricum acidum 30x | 59 | 10 | 0.88 | 0.31 | 0.127 |
| Kalium iodatum 30x | 59 | 10 | 0.93 | 0.30 | 0.363 |
| Kreosotum 30x | 52 | 10 | 0.92 | 0.29 | 0.329 |
| Lachesis 30x | 61 | 10 | 0.99 | 0.21 | 0.909 |
| Lycopodium 30x | 34 | 9 | 1.07 | 0.06 | 0.395 |
| Magnesium phosphoricum 30x | 58 | 10 | **1.20** | 0.12 | **0.011** |
| Nosode 30x | 21 | 10 | 0.91 | 0.09 | 0.252 |
| Phosphorus 30x | 57 | 10 | 1.09 | 0.23 | 0.250 |
| Plumbum metallicum 30x | 60 | 10 | 1.09 | 0.41 | 0.274 |
| Rhus toxicodendron 30x | 56 | 10 | 1.10 | 0.34 | 0.208 |
| Salycilicum acidum 30x | 54 | 10 | 1.11 | 0.28 | 0.157 |
| Secale cornutum 30x | 56 | 10 | 0.93 | 0.25 | 0.352 |
| Silicicum acidum 30x | 56 | 10 | 0.89 | 0.32 | 0.166 |
| Sulfur 30x x | 57 | 10 | **0.84** | 0.26 | **0.041** |
| Tabacum 30x | 57 | 10 | 1.06 | 0.28 | 0.436 |
| Zincum sulfuricum 30x | 59 | 10 | 0.92 | 0.24 | 0.341 |

Effects of homeopathic treatment on the infection rate of *A. thaliana* (number of colony forming units (cfu) of *P. syringae* bacteria per 0.5 cm$^2$ leaf area of *A. thaliana* plants, normalized to the control group set to 1). Statistical evaluation by LSD test (comparison with the pooled control group treated with water 1x). N = number of plants treated; SD = standard deviation. Statistically significant values are printed in bold.

The *A. thaliana/P. syringae* model system is quite time and labor consuming, and the effect size is smaller than we expected. We thus think that, after further optimization, this model might be interesting in order to identify the mode of action of homeopathic preparations in a complex model. However, for more basic questions of homeopathic fundamental research concerning production (e.g., suitable carrier materials, variation of potentization procedures, external disturbing factors), shelf life (relevance of production parameters and external influences), and application (effective potency levels, dosage, etc.), we recommend using simpler systems with smaller expenditures[25,27,29,30].

**TABLE 3**
***p* Values of the Global F Test of ANOVA for the Main Experiments Assessing the Effects of a Homeopathic Treatment and the Interaction of Treatment and Date of the Experiment on the Infection Rate of *A. thaliana***

| Preparation | Treatment | Interaction |
|---|---|---|
| Nosode 30x | 0.435 | 0.199 |
| Carbo vegetabilis 30x | 0.814 | 0.150 |
| Magnesium phosphoricum 30x | 0.154 | 0.704 |
| Biplantol SOS (original formulation) | **0.011** | 0.174 |
| Biplantol SOS 30x | 0.157 | 0.580 |

Statistically significant values in bold.

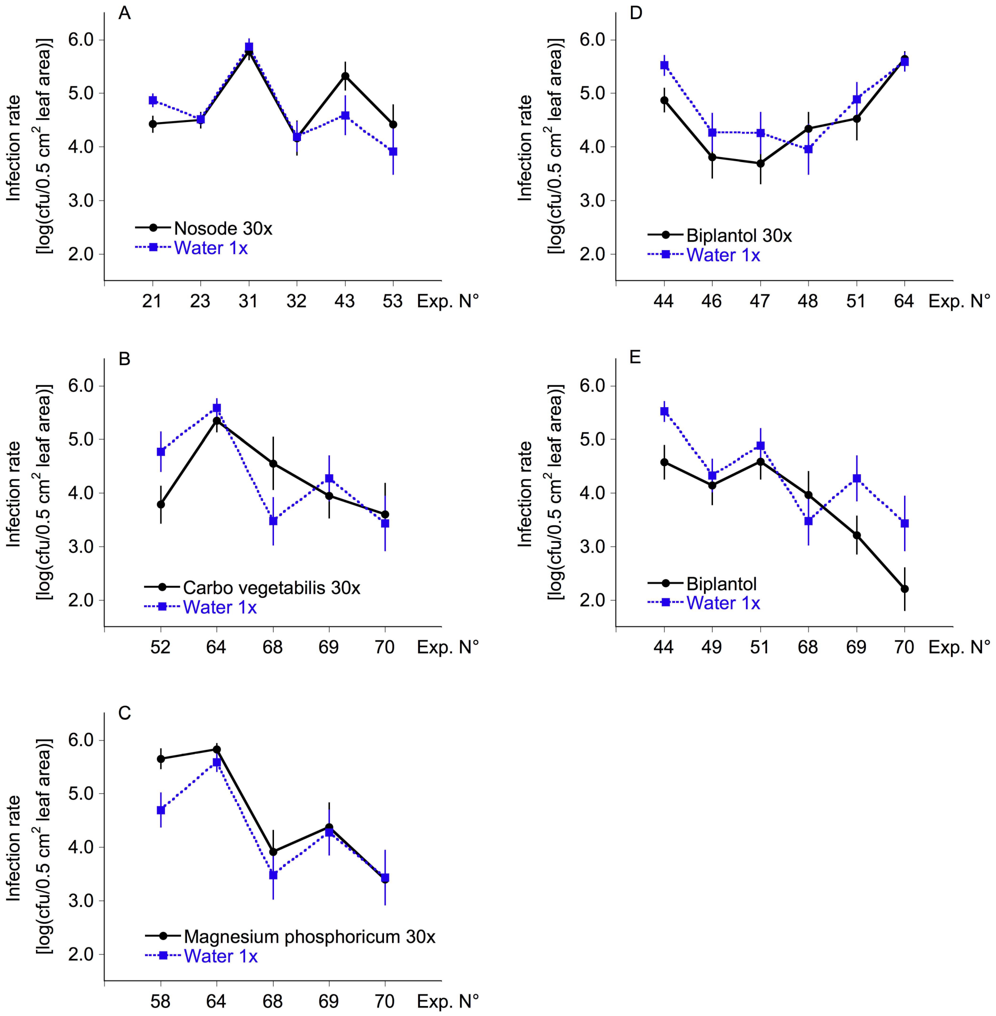

**FIGURE 1.** Number of bacteria of *P. syringae* in leaves of *A. thaliana* treated with different homeopathic preparations (A) Nosode 30x; (B) Carbo vegetabilis 30x; (C) Magnesium phosphoricum 30x; (D) Biplantol 30x; (E) Biplantol (in original formulation) or succussed water (1x) as control, as a function of independently performed experiments logarithm of the number of colony forming units per 0.5 $cm^2$ leaf area, mean ± standard error.

## CONCLUSION

In this investigation, we observed that a homeopathic complex remedy (Biplantol SOS in original formulation) revealed a significant therapeutic effect since its application reduced plant infection by decreasing the number of bacteria in the leaves of *A. thaliana*. As the efficiency of this homeopathic intervention was about 50% of the effect obtained with a nonhomeopathic plant immunity activator like Bion, it seems worth pursuing research into the effect of homeopathic preparations on the prevention and/or on the treatment of plant infections. We suppose that neither application nor contents of effective homeopathic formulations are optimized. Research should help to identify optimal application schemes concerning dosage (amount and frequency), timing (pre- or postinfection, daytime), and administration route (foliar spray, soil).

From a scientific point of view, it might be very interesting to identify the mode of action leading to reduced infection: Can homeopathic preparations activate SAR? If yes, what molecular pathways are involved? Are they similar or different compared to well-known SAR inducers? Or are homeopathic preparations able to act in a curative way, i.e., not by activation of SAR, but by inducing host responses after infection? After further optimization (i.e., increase of effect size), the *A. thaliana/P. syringae* model would be a well-suited model system for answering these types of questions.

For more basic questions of research into homeopathic preparations (e.g., effective potency levels, optimization of production procedures, shelf life), we recommend using simpler systems that are less time consuming and allow for a larger number of parameters to be tested in parallel.

Homeopathic treatment in agricultural practice may be of great interest due to the presumably reduced environmental impact. Since nothing is known about possible residues of homeopathic treatment on plants, we recommend careful consideration of the possibility of adverse effects before widespread application[13].

## ACKNOWLEDGMENTS

We are grateful to the Institute Hiscia in Arlesheim (Switzerland) for providing laboratory facilities; Dr. B. Mauch-Mani of Fribourg University (Switzerland) for the strain of *P. syringae* and helpful methodological information; Dr. M. Frei for homeopathic counseling; Dr. H. Flückiger for practical help during this project; Dr. P. Rossi of Neuchâtel University for discussions on microbiological aspects; Claudia Scherr and Tim Jäger for helpful comments on the manuscript; Phytomed AG in Burgdorf (Switzerland) and Bioplant GmbH in Konstanz (Germany) for providing the homeopathic preparations.

This work was supported by funds provided by the Asta Blumfeldt foundation in Dornach (Switzerland), GFBG foundation in Ittingen (Switzerland), Sampo foundation in Dornach (Switzerland), Similasan AG in Jonen (Switzerland), and Plantosan AG in Toffen (Switzerland). The sponsors had no influence whatsoever on study design, experimental implementation, data collection, statistical analysis, or manuscript preparation.

## References

1. Jacobs, J., Jonas, W.B., Jimenez-Perez, M., and Crothers, D. (2003) Homeopathy for childhood diarrhea: combined results and metaanalysis from three randomized, controlled clinical trials. *Pediatr. Infect. Dis. J.* **22,** 229–234.
2. Taylor, M.A., Reilly, D., Llewellyn-Jones, R.H., McSharry, C., and Aitchison, T.C. (2000) Randomised controlled trial of homoeopathy versus placebo in perennial allergic rhinitis with overview of four trial series. *BMJ* **321,** 471–476.
3. Barnes, J., Resch, K.L., and Ernst, E. (1997) Homeopathy for postoperative ileus? A meta-analysis. *J. Clin. Gastroenterol.* **25,** 628–633.
4. Jonas, W.B., Linde, K., and Ramirez, G. (2000) Homeopathy and rheumatic disease. *Rheum. Dis. Clin. North Am.* **26,** 117–123.
5. Lüdtke, R. and Wiesenauer, M. (1997) Eine Metaanalyse der homöopathischen Behandlung der Pollinosis mit Galphimia glauca. *Wien. Med. Wochenschr.* **147,** 323–327.

6. Vickers, A.J. and Smith, C. (2006) Homoeopathic oscillococcinum for preventing and treating influenza and influenza-like syndromes. *Cochrane Database Syst. Rev.* **3,** CD001957.
7. Linde, K., Jonas, W.B., Melchart, D., Worku, F., Wagner, H., and Eitel, F. (1994) Critical review and meta-analysis of serial agitated dilutions in experimental toxicology. *Hum. Exp. Toxicol*. **13,** 481–492.
8. Betti, L., Trebbi, G., Nani, D., Majewsky, V., Scherr, C., Jäger, T., and Baumgartner, S. (2008) Models with plants, microorganisms and viruses for basic research in homeopathy. In *Signals and Images*. Bonamin, L.V., Ed. Springer, Heidelberg. pp. 97–111.
9. Witt, C.M., Bluth, M., Albrecht, H., Weisshuhn, T.E., Baumgartner, S., and Willich, S.N. (2007) The in vitro evidence for an effect of high homeopathic potencies--a systematic review of the literature. *Complement. Ther. Med.* **15,** 128–138.
10. Baumgartner, S. (2005) Reproductions and reproducibility in homeopathy: dogma or tool? *J. Altern. Complement. Med.* **11,** 771–772.
11. Bastide, M. and Lagache, A. (1997) A communication process: a new paradigm applied to high-dilution effects on the living body. *Altern. Ther. Health Med.* **3,** 35–39.
12. Scofield, A.M. (1984) Homoeopathy and its potential role in agriculture – a critical review. *Biol. Agric. Hortic.* **2,** 1–50.
13. Baumgartner, S., Shah, D., Heusser, P., and Thurneysen, A. (2000) Homoeopathic dilutions: is there a potential for application in organic plant production? In *IFOAM 2000 – The World Grows Organic*. Alföldi, T., Lockeretz, W., and Niggli, U., Eds. vdf Hochschulverlag, Zürich. pp. 97–100.
14. Davis, K.R. (1993) Arabidopsis as a model plant system. In *Arabidopsis thaliana as a Model for Plant-Pathogen Interactions*. Davis, K.R. and Hammerschmidt, R., Eds. The American Phytopathological Society, St.Paul, MN. pp. 1–4.
15. Durrant, W.E. and Dong, X. (2004) Systemic acquired resistance. *Annu. Rev. Phytopathol*. **42,** 185–209.
16. Dong, X., Mindrinos, M., Davis, K.R., and Ausubel, F.M. (1991) Induction of Arabidopsis defense genes by virulent and avirulent Pseudomonas syringae strains and by a cloned avirulence gene. *Plant Cell* **3,** 61–72.
17. Whalen, M.C., Innes, R.W., Bent, A.F., and Staskawicz, B.J. (1991) Identification of Pseudomonas syringae pathogens of Arabidopsis and a bacterial locus determining avirulence on both Arabidopsis and soybean. *Plant Cell* **3,** 49–59.
18. Shirasu, K., Dixon, R.A., and Lamb, C. (1996) Signal transduction in plant immunity. *Curr. Opin. Immunol.* **8,** 3–7.
19. Agrios, G.N. (1997) *Plant Pathology.* Academic Press, San Diego, CA.
20. Huber, D.M. (1980) The role of mineral nutrition in defense. In *Plant Disease: An Advanced Treatise.* Horsfall, J.G. and Cowling, E.B., Eds. Academic Press, New York. pp. 381–406.
21. Baumgartner, S., Heusser, P., and Thurneysen, A. (1998) Methodological standards and problems in preclinical homoeopathic potency research. *Forsch. Komplementärmed*. **5,** 27–32.
22. Rikin, A. and Anderson, J.D. (1990) Rhythmic responses to hormones and herbicides in plants. In *Chronobiology: Its Role in Clinical Medicine, General Biology and Agriculture*. Hayes, D.K. et al., Eds. John Wiley & Sons, New York. pp. 859–903.
23. Kennedy, B.W. and Koukkari, W.L. (1987) Chronophytopathology. In *Advances in Chronobiology, Part A.* Pauly, J.E. and Scheving, L.E., Eds. Alan R. Liss, New York. pp. 95–103.
24. Carmer, S.G. and Swanson, M.R. (1973) An evaluation of ten pairwise multiple comparison procedures by Monte Carlo methods. *J. Am. Stat. Assoc*. **68,** 66–74.
25. Baumgartner, S., Thurneysen, A., and Heusser, P. (2004) Growth stimulation of dwarf peas (*Pisum sativum* L.) through homeopathic potencies of plant growth substances. *Forsch. Komplentarmed. Klass. Naturheilkd.* **11,** 281–292.
26. Baumgartner, S., Shah, D., Schaller, J., Kämpfer, U., Thurneysen, A., and Heusser, P. (2008) Reproducibility of dwarf pea shoot growth stimulation by homeopathic potencies of gibberellic acid. *Complement. Ther. Med.* **16,** 183–191.
27. Scherr, C., Simon, M., Spranger, J., and Baumgartner, S. (2007) Duckweed (Lemna gibba L.) as a test organism for homeopathic potencies. *J. Altern. Complement. Med.* **13,** 931–938.
28. Betti, L., Lazzarato, L., Trebbi, G., Brizzi, M., Calzoni, G.L., Borghini, F., and Nani, D. (2003) Effects of homeopathic arsenic on tobacco plant resistance to tobacco mosaic virus. Theoretical suggestions about system variability, based on a large experimental data set. *Homeopathy* **92,** 195–202.
29. Brizzi, M., Nani, D., Peruzzi, M., and Betti, L. (2000) Statistical analysis of the effect of high dilutions of arsenic in a large dataset from a wheat germination model. *Br. Homoeopath. J*. **89,** 63–67.
30. Pongratz, W., Nograsek, A., and Endler, P.C. (1998) Highly diluted agitated silver nitrate and wheat seedling development. In *Fundamental Research in Ultra High Dilution and Homoeopathy*. Schulte, J. and Endler, P.C., Eds. Kluwer Academic, Dordrecht. pp. 143–154.
